# Upright 0.5 T open MR defaecating proctography proof of concept study: the inter- and intra-rater variability of pelvic floor measures using seated proctography

**DOI:** 10.1007/s10334-025-01296-6

**Published:** 2025-09-13

**Authors:** Rashed Sobhan, Paul M Glover, Penny A Gowland, Rahul Munyal, Olivier E Mougin, Christopher G D Clarke

**Affiliations:** 1 Sir Peter Mansfield Imaging Centre, University of Nottingham, Nottingham, NG7 2RD UK; 2 Department of Radiology, Nottingham University Hospitals NHS Trust, Nottingham, UK; 3 NIHR Nottingham Biomedical Research Centre, Nottingham University Hospitals NHS Trust, Nottingham, United Kingdom

**Keywords:** Upright proctography, Pelvic floor imaging, Open magnet, Repeatability, Reproducibility

## Abstract

**Objective:**

We tested the feasibility, data quality, and reliability of an upright magnetic resonance defaecating proctography (uMRDP) technique using an Open 0.5 T ASG MRI scanner.

**Materials and methods:**

Eight healthy volunteers (2 males) performed seated defaecation on a purpose-built radio-frequency commode coil in an Open scanner. An optimised T2-weighted HASTE sequence captured dynamic changes during all three phases of the Kegel manoeuvre. Inter- and intra-rater variability was measured from the pelvic floor (PF) metrices (M-line, H-line, distances of landmarks) extracted by two radiologists.

**Results:**

All relevant PF landmarks could be identified and metrices were extracted with acceptable inter- and intra-rater variability. Intra-rater variation was marginal, with relative absolute differences ranging from 5 to 21% and 3.2–44%. Inter-rater variability was reported using correlation and Bland-Altman plots. Correlation between raters was satisfactory, with *r*^2^ > 0.93, and bias ranged from − 1.8 to 0.65 mm. Moreover, the limit of agreement in the Bland-Altman plot ranged from 5.8 to 20.4 mm, indicating satisfactory precision.

**Discussion:**

The proposed uMRDP technique can be used as a feasible and reliable alternative to supine MRDP, without the necessity of gadolinium injection and bowel preparation. It can capture defaecation in a regular seated posture and can provide information complementary to standard-of-care fluoroscopic proctography for clinicians.

**Supplementary Information:**

The online version contains supplementary material available at 10.1007/s10334-025-01296-6.

## Introduction

Defaecation disorders, such as intussusception, rectocele, enterocele and pelvic organ prolapse, severely impact the quality of life of sufferers and incur a significant economic burden on health services [1]. Due to age and prior history of multiple instrumented or vaginal deliveries, nearly 50% of multiparous women aged more than 50 years suffer from some form of pelvic floor disorder [2]. In the UK, a survey by the Royal College of Obstetricians and Gynaecologists on two thousand women reported that over 60% of women have at least one symptom of poor pelvic floor health, with 69% not having spoken about their pelvic floor health to anyone in the NHS [3]. In the USA, nearly 25% of women receive a diagnosis of pelvic floor disorder, and approximately 200,000 women undergo surgery every year [4]. Patients typically present with complaints/symptoms of faecal incontinence, pain during defaecation, chronic constipation, abnormal urination, sphincter defects, or sexual dysfunction [1, 4, 5].

Imaging of the rectum is an important component of the conventional clinical assessment of pelvic floor disorder and subsequent surgical planning. The two main techniques used routinely are fluoroscopic proctography or supine magnetic resonance imaging (MRI) defaecography. Fluoroscopic proctography involves collecting a series of X-rays during defaecation after administering barium contrast per rectum (and often orally to outline the small bowel). As X-ray imaging is a planar imaging tool, fluoroscopic proctography cannot image all three pelvic floor compartments at one time; it only images the luminal aspect of the bowel wall [6]. Thus, it is less sensitive at detecting abnormalities in the anterior pelvic compartment e.g., the urinary bladder, caused by general pelvic floor weakness [5]. Moreover, it involves a significant level of ionising radiation exposure [5, 7] for women of child-bearing age [1] with a mean effective dose of 4.9 mSv [8].

Contrary to X-ray-based techniques, MRI involves no ionising radiation and, therefore, has no potential detrimental effects to future health. Moreover, it allows superior temporal and spatial resolution as well as imaging in multiple planes [8] with superior contrast to enable better delineation of the pelvic floor anatomy. However, conventional supine MR-based defaecating proctography (sMRDP) involves patients attempting to push out a gel from their rectums whilst lying down; patients find it difficult and uncomfortable to carry out the voiding phase, which reveals the most clinically relevant information [1]. In addition, due to this abnormal posture, the process fails to mimic the normal structural or functional changes of the pelvic floor during defaecation [9], partially limiting the clinical/diagnostic value of sMRDP. For example, fluoroscopic proctography is more sensitive than MRDP in detecting rectal intussusception, a condition where the bowel wall folds in on itself during defaecation [9]. A study has shown that only 50% of patients were able to push out an artificial stool when lying down compared to 80% when sitting up [10].

Upright MRDP (uMRDP) with an open configuration magnet allows patients to defaecate in their regular seated posture. Thus, it mitigates the limitations of sMRDP and enables clinicians to visualise anorectal morphology and functional changes of the complete pelvic floor while patients are defaecating in their typical upright posture.

Many previous studies have investigated the prospects of uMRDP and compared its utility to fluoroscopic proctography, but not without limitations related to patient comfort, image quality, and/or resolution for capturing dynamic details. For example, many used T_1_-weighted imaging to suppress water contrast, which results in poor imaging of the bladder and bowel. They enhanced contrast by mixing gadolinium-based contrast agent (GBCA) with rectal paste (i.e., ultrasound gel, mashed potatoes, etc.) [1, 5–8], but gadolinium contrast carries the risks of allergic reaction and involves the additional cost and environmental impact of GBCA [11]. Many studies have also used urethral catheters or placed markers in the vagina and/or rectum to optimise their data quality [1, 8, 12–15], but such methods are invasive, may be uncomfortable, and could increase the risk of infection. Moreover, the existing radio-frequency (RF) coils for typical uMRDP imaging might not give dedicated coverage to the pelvic floor areas, limiting image quality. Coils strapped around the pelvis might cause discomfort and intervene with the natural manoeuvres during defaecation [7, 8]. Therefore, alternative imaging sequences and a comfortable RF coil structure with suitable coverage should improve the efficacy of the technique.

In this study, we use a 0.5 T ASG Open MR scanner with a purpose-built RF commode coil and optimised T2-w acquisition protocol to propose a safe and comfortable method of performing uMRDP in the sitting position. The commode system with integrated RF coils substitutes the strapping of coils around the pelvis and ensures comfort as well as uninterrupted pelvic floor movement during defaecation. The T2-w dynamic sequence aims to provide sufficient contrast without invasive GBCA injection into the vagina, urinary bladder or small bowel. Our aim is three-fold: first, to investigate the feasibility of the equipment and sequences for imaging seated defaecation in healthy volunteers; second, to assess the quality of the dynamic data for extracting the conventional anatomical and functional metrices used for clinical assessments; finally, to evaluate the inter-rater and intra-rater variability in pelvic floor metrices, as extracted by two expert Radiologists.

## Materials and methods

### Participants and experimental set-up

After approval from the local ethics committee (FMHS 215-0223), participants were recruited via advertisement and written consent was received before scanning. Eight healthy volunteers (2 males; median age 23 years, ranging from 21 to 44 years) with no history of pelvic floor abnormalities, bowel disorders, neurological or psychiatric conditions were scanned inside a 56 cm lateral gap vertical open 0.5 T scanner (MROpen, Paramed, Genoa, Italy) at University of Nottingham, UK in between September 2023 and May 2024. The Open MRI scanner allows scanning in seated, supine, prone, and standing positions and can operate with a maximum gradient strength of 20 mT/m and a maximum slew rate of 33 mT/m/ms in all three axes.

**Fig. 1:**
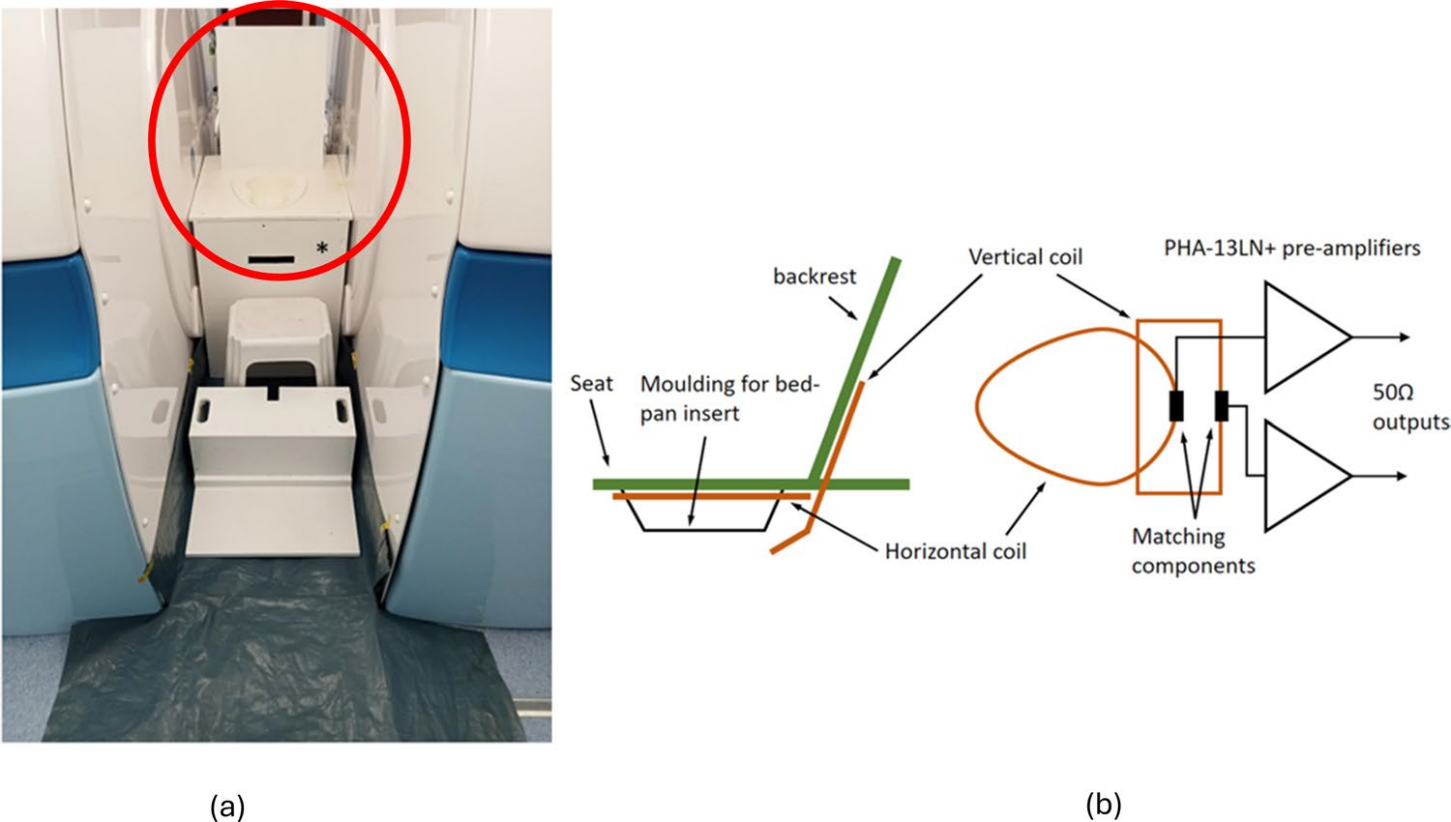
**a** Experimental setup for upright MR defaecating proctography. The purpose-built RF commode coil is circled in red; **b** the schematic diagram showing the components of the RF commode coil

No prior bowel preparation was necessary for participants. Privacy was ensured with opaque screens around the scanner and clinical room door; necessary communication during scans was achieved using an intercom. To ensure patient comfort as well as to obtain optimal anatomical coverage, we built a dedicated RF coil with a commode moulded on it, instead of using strapped coils (Fig. 1a). Figure 1b provides a schematic diagram of the components of the commode coil. The two coils of the RF commode were arranged at 105°, but with a small bend in the vertical/back coil to null the coupling between coils. The horizontal coil shape was determined by the outline of the bedpan cutout moulding, and the vertical coil was  oblong. The single-turn coils were made from 4 mm diameter copper wire, tuned, and matched to 50 ohms. The coils were passively switched off during transmit pulses using crossed diodes. The pre-amplifiers were low-noise Mini-Circuits PHA-13LN+ devices.

### Image acquisition

For the first half of the scanning, before injecting any ultrasound (US) gel, participants were asked to sit on the commode coil in their regular defaecation posture, leaning on a hand-rail to increase stability and reduce movement artefacts. Static images were acquired using a sagittal fast spin-echo (FSE) sequence and an axial FSE single-slice sequence. A slice passing through the midline in the sagittal plane was selected for dynamic imaging. Dynamic images were acquired every 1.5 s during different stages of defaecation. To optimise the dynamic acquisition protocol, several half-Fourier acquisition half-Fourier single-shot turbo spin echo (HASTE) sequences were run while participants were instructed by the Radiologist to rest, clench, and push (i.e., strain) their pelvic floor without performing any defaecation. Table 1 gives the parameters of the sequences compared. The Radiologist then selected the most appropriate dynamic sequence with suitable contrast and resolution to be used for the latter half of the scanning with rectum filling.

**Table 1:** Imaging parameters for static and dynamic imaging

Sequence	TR/TE (ms)	FA (°)	FOV (mm^2^)	Resolution (mm^2^)	Slice thickness (mm)	Comment
SAG FSE T2	4865/104	90/180	471 × 350	1.40 × 1.40	4	Two excitations to increase SNR
AX FSE T2	800/104	90/180	634 × 350	1.40 × 1.40	5.5	PCL used for planning the slice
HASTE-OPTIM-10 mm	1500/150	90/160	471 × 350	2.50 × 2.30	10	Higher slice thickness to explore more SNR (*n* = 2)
HASTE-OPTIM-7 mm	1500/150	90/160	471 × 350	2.50 × 2.30	7	Reduction of the slice thickness to increase resolution (*n* = 5)
HASTE-OPTIM-5 mm	1520/100	90/160	471 × 350	2.40 × 1.30	5	Further reduction of slice thickness to increase resolution (*n* = 1)

Baseline FSEs and HASTE-OPTIM-10 mm and HASTE-OPTIM-7 mm were acquired for comparison for all subjects; ‘*n*’ in the comment column presents the number of subjects for whom the sequence was deemed optimal by the Radiologists. For one subject, neither 7 nor 10 mm was optimal, but 5 mm was satisfactory

*PCL* pubo-coccygeal line, *FSE* fast spin echo, *TR* repetition time, *TE* echo time, *FOV* field of view, *FA* flip angle, *SNR* signal-to-noise ratio, *HASTE* half Fourier single-shot turbo spin echo, *mm* millimetres

After the first half, participants were taken to a clinical room and approximately 150 ml US gel was injected into the rectum while participants lay in the left-lateral position. Participants were then asked to sit back on the commode coil in complete privacy. After acquiring the structural scans, dynamic data were collected with the pre-selected optimised dynamic sequence while participants performed Kegel manoeuvres expelling the gel under the Radiologist's instructions (i.e., ‘rest’, ‘clench’, ‘push’). If all the US were not evacuated in one run, the next optimal dynamic sequence was run whilst the participant was again asked to perform the Kegel manoeuvre in synchrony to the Radiologist’s command. The overall scan time for each participant was under 1 h.

### Image analysis

For each stage of the Kegel manoeuvre—namely, rest, clench, and defaecation—two Radiologists (CC and RM) with 8 and 4 years of consultant experience, respectively, extracted the following pelvic floor metrices:

1. Pubo-coccygeal line (PCL): the line drawn on the sagittal plane that extends from the inferior border of the pubic symphysis (PS) to the last visible coccygeal joint [1]. It defines the base of the pelvic floor [1] and acts as the reference line for pelvic floor disorder grading. The spatial locations of other organ-specific reference points at rest and during different stages of manoeuvres are defined as the perpendicular distance from PCL.
2. H-line (hiatal width): the line from the inferior border of PS to the posterior border of the pubo-rectalis muscle. This line acts as an index of widening of the pubo-rectal hiatus and used to measure the anteroposterior diameter of the pelvic hiatus [1, 4].
3. M-line (hiatal descent): the line perpendicular to the PCL from the posterior-most border of H-line. This line gives the index of pelvic floor descent.
4. The distance between the PCL and the lowest recognizable part of the urinary bladder, posterior vaginal fornix, rectum, and small intestine.
5. Anorectal angle (ARA): ARA is the angle at the intersection between line tangent to the posterior wall of the rectum and a line parallel to the axis of the anal canal and the anorectal junction. ARA represents the function of the pubo-rectal muscle. An increase in ARA collectively reflects an increase in the intra-abdominal pressure.

Each radiologist extracted the above metrics twice, separated by at least a one-month time interval. Before making these measurements, both raters reached a consensus on how they defined the two endpoints of PCL from the dynamic data. As most metrices were calculated as perpendicular distances from PCL, subjective choice of PCL end-points can introduce significant inter-rater variability in those metrices. No pelvic floor disorder grading was performed as the cohort was healthy and our primary target was to assess data quality and consistency in measurements between multiple raters.

### Inter- and intra-rater variability analysis

All statistical analysis was performed using MATLAB (R2023a, Natick, MA). Intra-rater variability was assessed by quantifying the relative absolute difference (RAD) between two measurements from the same Radiologist. The mean and interquartile range (IQR) of RADs were reported for each pelvic floor metric. Inter-rater agreement was reported using correlation and illustrated by a Bland–Altman plot for each metric [16]. From the correlation plots, the following parameters are reported: sum of squared error for the linear regression fit (SSE); *r*^2^: Pearson correlation r-value squared; linear fit equation with slope and intercept. From the Bland–Altman plot, bias and coefficient of variation were reported along with reproducibility coefficient estimate based on IQR (RPC_np_ = 1.45 × IQR) as all metrices failed the Kolmogorov–Smirnov normality test (with ks > 0.05). ARA was interpreted separately as it is an angular metric, as opposed to the other length metrices.

## Results

### Quality of dynamic data

**Fig. 2:**
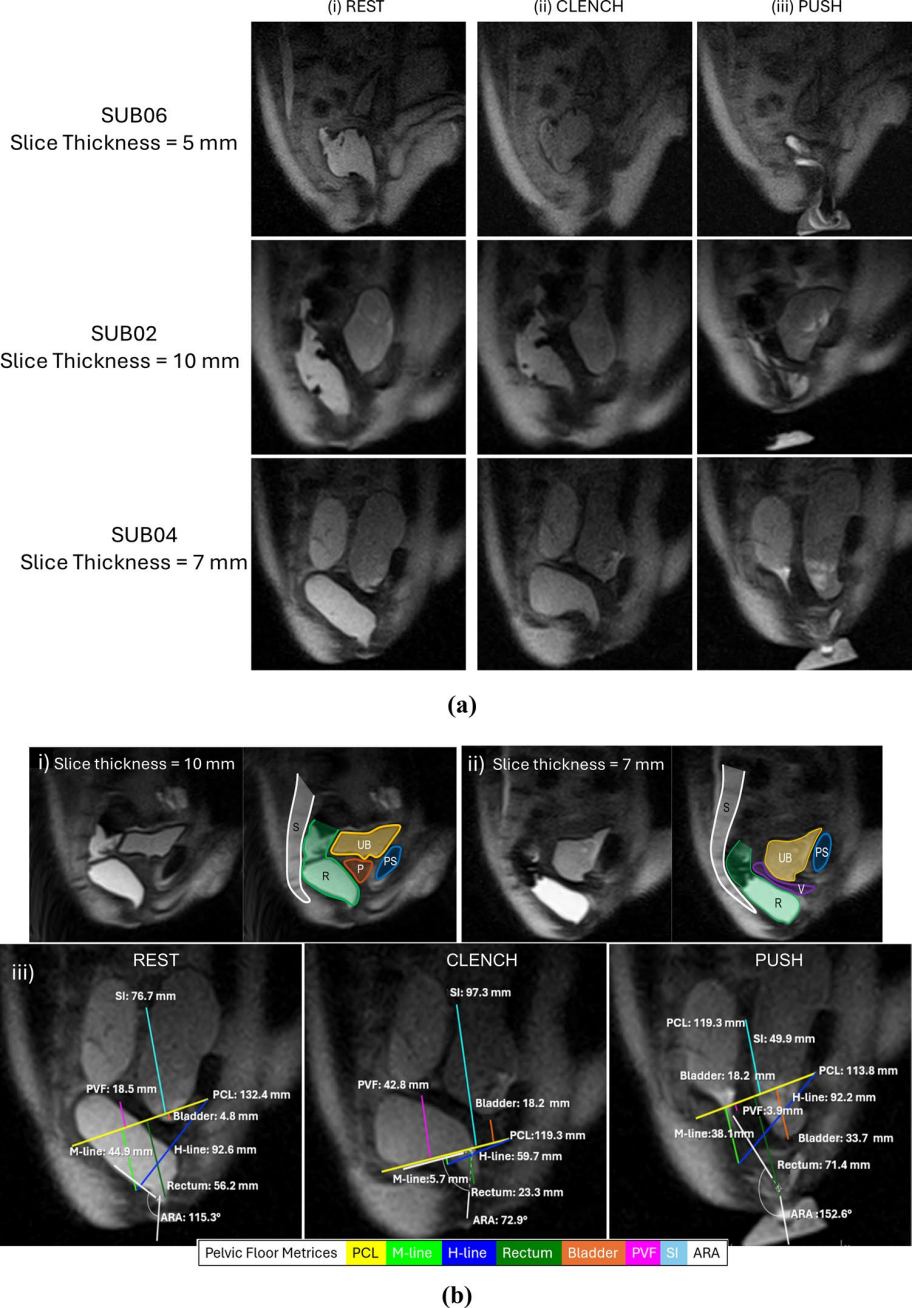
**a** Pelvic floor images captured during (i) rest, (ii) clench, and (iii) push stages of seated defaecation for three participants using HASTE sequences with three different slice thicknesses, showing the quality of data acquired. **b** Anatomy of a male participant (i) and female participant (ii) demonstrating the urinary bladder (UB), Rectum (R), Pubic symphysis (PS), Prostate (P), Vagina (V), Sacrum and coccyx (S). (iii) Pelvic floor metrices extracted at rest, clench, and push stages of defaecation for a dynamic image with 7 mm slice thickness (with colour keys). Abbreviations/definitions: pubococcygeal line (PCL), the descent of levator plate (M-line); the length of the hiatus (H-line); posterior vaginal fornix (PVF); small intestine (SI); anorectal angle (ARA). Note: Larger format images with measured pelvic floor metrices for **a** are provided in the supplementary document

Figure 2a shows three time points from three dynamic HASTE data corresponding to each stage of the Kegel manoeuvre. The sequence had sufficient temporal resolution of 1.5 s to capture the changes in the H-line, M-line, ARA, and other metrices during each stage of defaecation. As can be seen from Fig. 2bi–ii, with the different HASTE sequences with different slice thicknesses, the pelvic floor organs and specific landmarks can be identified. Regardless of slice thickness, the bladder, pubic symphysis, posterior vaginal fornix, vagina and cervix, rectum can be located, and their lowest part can be identified to make the measurements. For most of the participants (5 out of 8), the Radiologists preferred the HASTE sequence with 7 mm slice thickness as the optimal compromise between signal-to-noise ratio and resolution. Figure 2biii shows pelvic floor metrices extracted during three stages of Kegel Maneuver for a participant whose dynamic defaecation data were acquired using optimised HASTE. The supplementary documents contain gifs showing dynamic MRI of defaecation for the three participants in Fig. 2a, along with larger versions of images with pelvic floor metrices extracted from Fig. 2a participants for better visualisation and legibility of annotations.

### Inter- and intra-rater variability

**Fig. 3:**
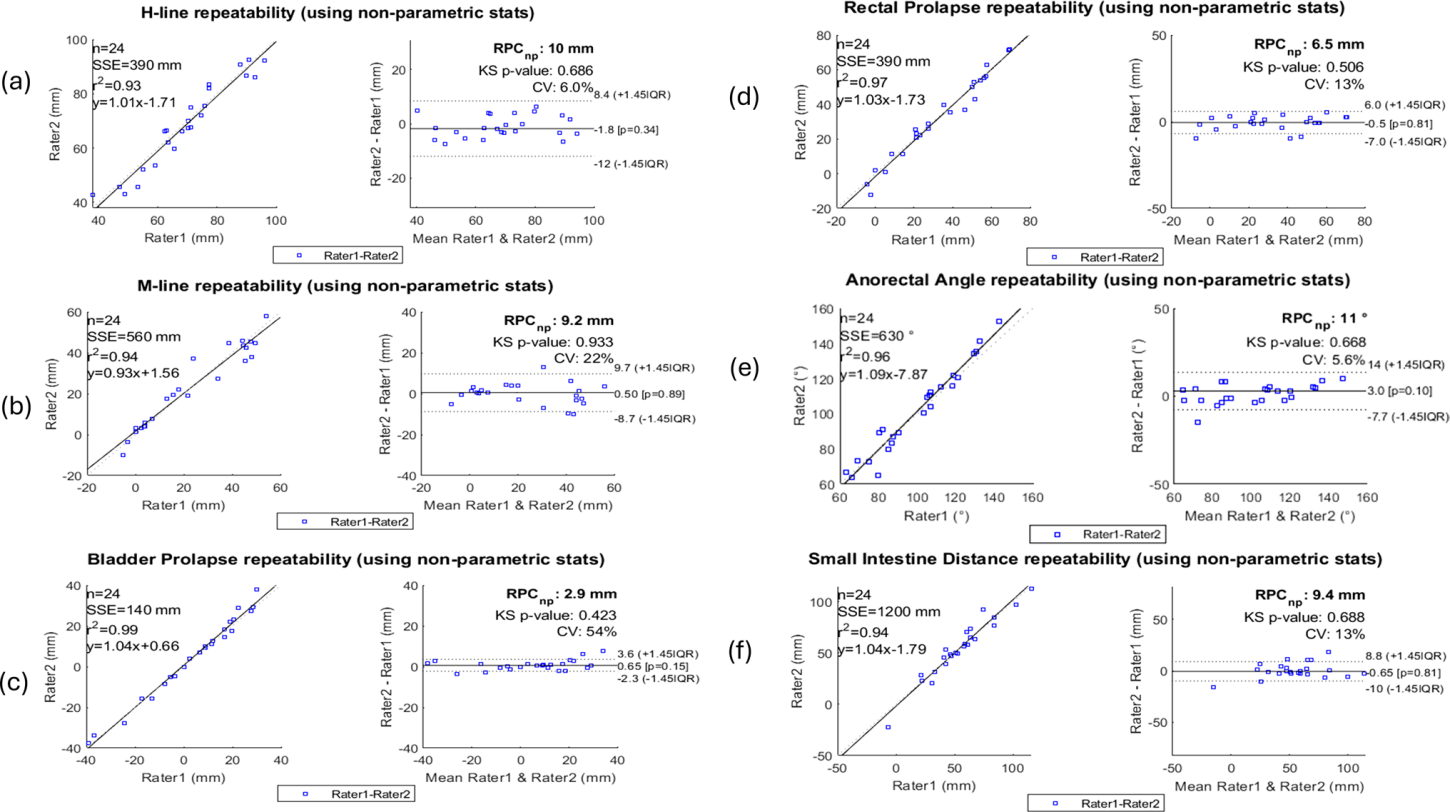
Correlation and Bland–Altman plots showing the inter-rater agreement in pelvic floor measures. Correlation plot: ‘SSE’—sum of squared error for the linear regression fit; ‘*r*^2^’—Pearson r-value squared; ‘eq’—linear fit equation with slope and intercept; Bland–Altman plot: ‘RPC_np_’—reproducibility coefficient (RPC) estimate based on interquartile range (for non-parametric statistics); ‘ks’—Kolmogorov–Smirnov test (ks < 0.05 rejects the null hypothesis that the distribution is normal); ‘CV’—coefficient of variation (SD of mean values in %)

Table 2 summarises intra- and inter-rater variability parameters and Fig. 3 gives the correlation and Bland–Altman plot for the metrices. Median of intra-rater variability parameter (RAD) ranged from 5 to 21% and 3.2 to 44% for two raters; the differences in distance measures are small in comparison to the relative size of the pelvic floor organs, which suggests good intra-rater repeatability using uMRDP acquisition.

**Table 2:** Intra- and inter-rater variability parameters for the pelvic floor metrices

Measures	H-line (mm)	M-line (mm)	Bladder prolapse (mm)	Rectum prolapse (mm)	ARA (°)	SI distance (mm)	PVF distance (mm)
Intra-rater variability							
Median (IQR) of Rater 1 RAD (%)	8.9 (8.6)	18.2 (46.4)	7 (22)	8.9 (11.7)	5 (6.4)	20.9 (29.5)	16.6 (37.6)
Median (IQR) of Rater 2 RAD (%)	7.8 (12.5)	44.3 (79.5)	3.2 (27.7)	14.6 (17.3)	13.1 (10.4)	28.9 (43.3)	15.3 (29)
Inter-rater variability							
Pearson *r*^2^ (slope of correlation line)	0.93 (1.01)	0.94 (0.93)	0.99 (1.04)	0.97 (1.03)	0.96 (1.09)	0.94 (1.04)	0.96 (1.06)
Bias	− 1.8	0.50	0.65	− 0.5	3	− 0.65	− 2.0
LOA	20.4	18.4	5.8	13	22	18.8	9.8
CV (%)	6	22	54	13	5.6	13	21

*RAD* relative absolute difference, *IQR* interquartile range, *ARA* anorectal angle, *SI* small intestine, *PVF* posterior vaginal fornix, *IQR* interquartile range, *LOA* limit of agreement (2 × RPC_np_), *CV* coefficient of variation

‘RPC_np_’—reproducibility coefficient estimate based on interquartile range (non-parametric statistics) where RPC_np_ = 1.45 × IQR ~ RPC (if distribution of differences is normal); ‘CV’—coefficient of variation (standard deviation of mean values in %); Pearson *r*^2^ is the square of correlation coefficient, describing agreement between the raters

The correlation plots of the length-based metrices show a good linear correlation between the raters, with *r*^2^ ranging from 0.93 to 0.99. The slopes of the linear fits range between 0.93 and 1.09. For ARA, the *r*^2^ was 0.96 with a slope of the linear fit of 1.1; this suggests good agreement between the raters.

From the Bland–Altman plots of the metrices, it is evident that the mean of the differences for each metric is close to zero (ranging from − 0.5 to 0.65 mm), suggesting marginal bias between the two raters. For all the metrices, the differences are scattered randomly around the mean with no visible trend, indicating consistent, non-systematic difference across measurements. The 95% confidence interval of the differences, i.e., the limit of agreement (2 × RPC_np_), is between 5.8 mm (Bladder Prolapse) and 20.4 mm (H-line), which suggests that raters have extracted metrices with satisfactory precision. Further, the coefficient of variation values was generally low, except for bladder prolapse.

The Bland–Altman plot for ARA shows a small bias of 3°, suggesting good agreement between the two raters. As with the other metrices, the differences are also randomly scattered around the mean with no visible trend. The 95% confidence interval was 22°, which suggests satisfactory precision. Finally, the coefficient of variation is 0.056, which provides further evidence of moderate precision.

## Discussion

This proof of concept study investigates the feasibility of an upright MRDP setup and explores the quality of the data acquired using a purpose-built RF coil and optimised T2-w protocol to extract pelvic floor metrices without the use of GBCAs. Moreover, the inter- and intra-rater variability in the pelvic floor metrices were estimated to determine whether Radiologists can reliably extract conventional and complementary metrices from healthy pelvic floor images using the proposed uMRDP method. This study was performed on healthy volunteers to optimise the participant management procedures, set-up, protocol, and equipment in terms of safety, comfort and convenience, so that pelvic floor disorder patients can be scanned with minimal disturbance in future.

The dynamic data is of satisfactory quality for identifying relevant pelvic floor organs, landmarks, and locations of organ-specific reference points. The agreement between raters was satisfactory for all pelvic floor metrices; also, the intra-rater variation was marginal (in the order of millimetres), indicating satisfactory repeatability and reproducibility of the proposed uMRDP technique. In contrast to previous reports of vertical defaecography MRI [1, 5, 7, 8, 15], our approach avoided invasive bladder catheterisation and our T2-w sequence avoided the need to use GBCA with T1w sequences whilst achieving an acceptable balance between the spatial and temporal resolution and signal-to-noise ratio and providing sufficient soft-tissue-contrast to allow radiologists to delineate landmarks, such as the rectal wall, coccygeal joints, other organ borders, and reference points. The volume and texture of rectum-injected US gel somewhat mimicked those of faecal matter and provided sufficient contrast to visualise faecal movement. In addition, unlike previous studies, this method needed no bowel preparation or invasive catheterisation procedure to distend the urinary bladder or vagina with GBCA [1, 15].

Furthermore, the purpose-built RF coil commode system allowed participants to recreate their natural sitting defaecation posture inside the open MRI, without the additional discomfort of having a coil strapped around their pelvis [7]. The relatively normal sitting arrangement and complete privacy provided by the scanner bore and minimal screening provided participants with a setup that was more similar to standard fluoroscopic proctography.

Both raters extracted the metrices with sufficient repeatability and reproducibility. Defining the end-points of PCL was an essential initial step as most of the metrices depend on the perpendicular distance from PCL, and inconsistent definition causes higher variability. We used Bland–Altman plots for illustrating inter-rater agreement. Although these plots are often used for making comparison between two methods of measurement, they are also used in literature as graphical tools to represent inter-rater agreement for continuous variables [17–19]. As only healthy participants were imaged, metrices could not be categorised/classified into pelvic floor disorder grades and, therefore, further reliability measures such as inter-class correlation coefficient or Cohen’s kappa were not used.

This work has limitations. First, the small sample size limits the power of the statistical analysis or generalisability of the results. Clearly, it is not appropriate to extend this intrusive study to many healthy subjects, but this data provides initial evidence confirming the feasibility of our approach, and also provides some evidence on the variability of the measurements. Thus, it can form the basis for a study on a broader cohort of patients, which in itself might lead to a formal trial. As it was a feasibility study, a detailed power calculation was not possible, but this data will assist in powering future studies in patient populations. Secondly, since only healthy volunteers were scanned, the parameters of the optimal T_2_-w sequence might need some modification for the pelvic floor disorder patient cohort. For example, a different slice thickness might be suitable for specific pelvic floor disorders. This study aimed at establishing the methodology for such patient studies; it defines a robust protocol with good quality data that can be used on a pelvic floor disorder cohort to compare fluoroscopic proctography and uMRDP in terms of patient experience, acceptance, image quality, and diagnostic accuracy. Extension of the study to the pelvic floor disorder cohort will be more informative in a clinical context. Third, the necessity of a prior consensus limits the immediate scalability of the study, although this should be addressable with a clearly defined published consensus to keep the inter-rater variability within an acceptable range. Fourth, no study to our knowledge suggests an acceptable range of inter- and intra-rater variability for MRDP. Thus, we cannot suggest whether the variability is acceptable or not. However, as the bias is near zero, the limit of agreement is small, and the correlation between raters is sufficiently linear, we can suggest that the proposed uMRDP could be repeatable and reproducible across centres.

For pelvic floor disorder diagnosis and treatment planning, uMRDP with an associated commode coil and optimal sequence selection could give complementary information to standard-of-care fluoroscopic proctography, including all compartments of the pelvic floor. For example, complete pelvic coverage and high soft-tissue contrast will allow radiologists to understand the degree of intussusception and/or rectal prolapse. Surgeons could be more certain as to whether any enterocele or sigmoidocele is co-existing with a rectocele (thus instigating or suggesting a transabdominal approach). Further studies evaluating patient acceptability and comparison between uMRDP and fluoroscopic proctography are required.

## Conclusion

This proof-of-concept study demonstrates that dynamic upright MRDP can be performed using a dedicated commode coil, and without using GBCA or catheterisation as an alternative to supine MRDP, which is a poor model for normal defaecation. The variability analysis suggests that the quality of uMRDP dynamic data is good enough for Radiologists to identify the associated pelvic floor landmarks and extract metrices with acceptable inter- and intra-rater variability. For centres with access to upright scanners, this technique could provide complementary information to fluoroscopic proctography for the clinicians.

## Supplementary Information

Below is the link to the electronic supplementary material.

Supplementary file1 (PPTX 10611 kb)

## Data Availability

Data sets generated during the current study are available from the corresponding author on reasonable request.Ethical approval was granted by the local ethics committee (FMHS 215-0223). Each participant received a comprehensive description of the study, including possible inconveniences, and gave written informed consent before participation. The authors declare no potential conflicts of interest with respect to the research, authorship, and/or publication of this article.

## Abbreviations

ARA: Anorectal angle
CV: Coefficient of variation
FA: Flip angle
FOV: Field of view
FSE: Fast spin echo
GBCA: Gadolinium-based contrast agent
HASTE: Half Fourier single-shot turbo spin echo
IQR: Interquartile range
LOA: Limit of agreement
MRDP: Magnetic resonance defaecating proctography
MRI: Magnetic resonance imaging
PCL: Pubo-coccygeal line
PS: Pubic symphysis
PVF: Posterior vaginal fornix
RAD: Relative absolute difference
RF: Radio-frequency
RPC: Reproducibility coefficient
SD: Standard deviation
SI: Small intestine
SNR: Signal-to-noise ratio
SSE: Sum of squared error
TE: Echo time
TR: Repetition time
uMRDP: Upright magnetic resonance defaecating proctography
US: Ultrasound
