# Supplementary material for: Upright 0.5 T open MR defaecating proctography proof of concept study: the inter- and intra-rater variability of pelvic floor measures using seated proctography
Source: MAGMA. 2025 Sep 13;39(1):157–65. doi: 10.1007/s10334-025-01296-6 (PMC12901193; doi:10.1007/s10334-025-01296-6)
Supplement: Supplementary file 1 — Supplementary file1 (PPTX 10611 kb) [file 10334_2025_1296_MOESM1_ESM.pptx]

## Slide 1
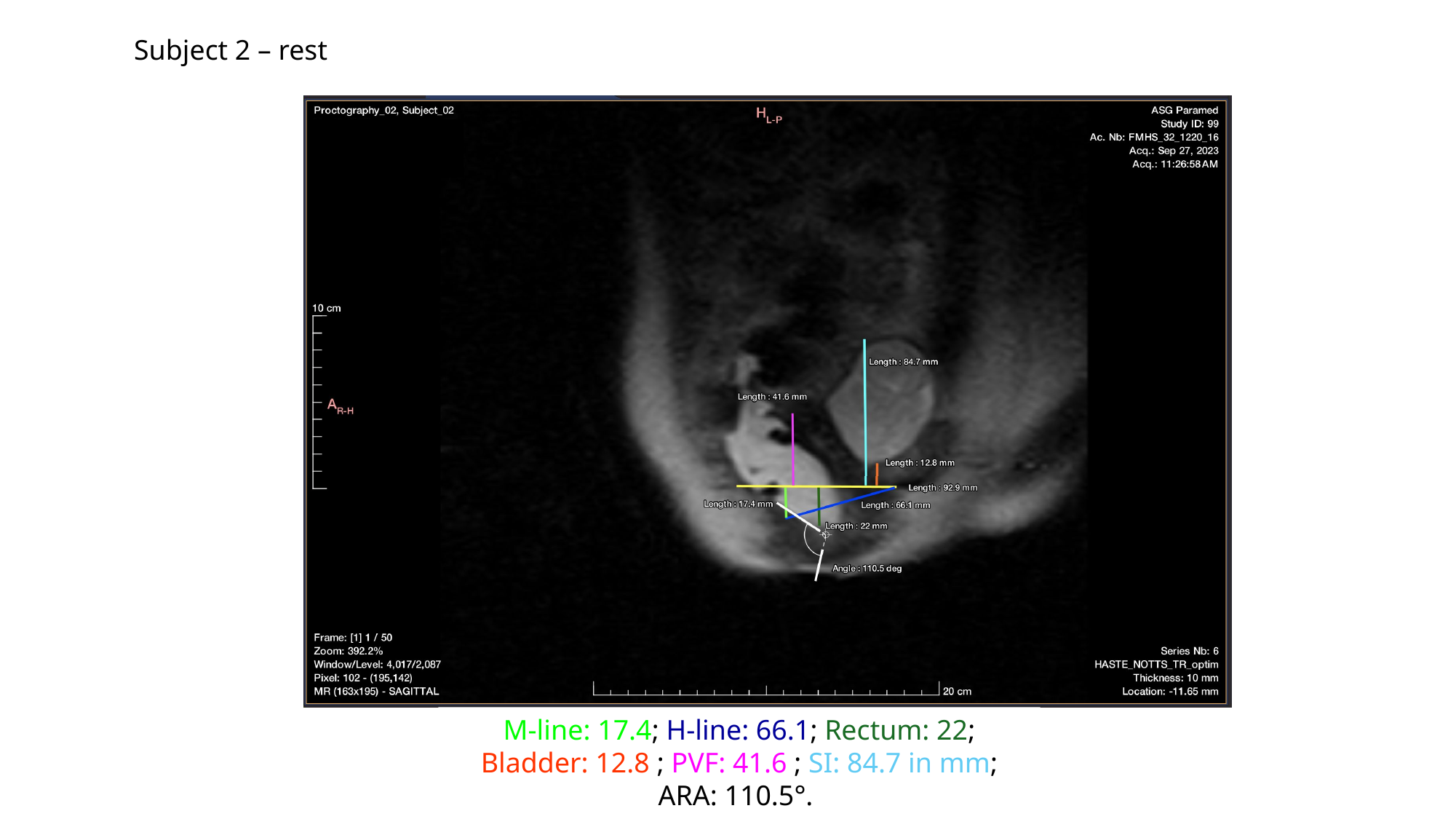

Subject 2 – rest
M-line: 17.4; H-line: 66.1; Rectum: 22; Bladder: 12.8 ; PVF: 41.6 ; SI: 84.7 in mm; ARA: 110.5°.

## Slide 2
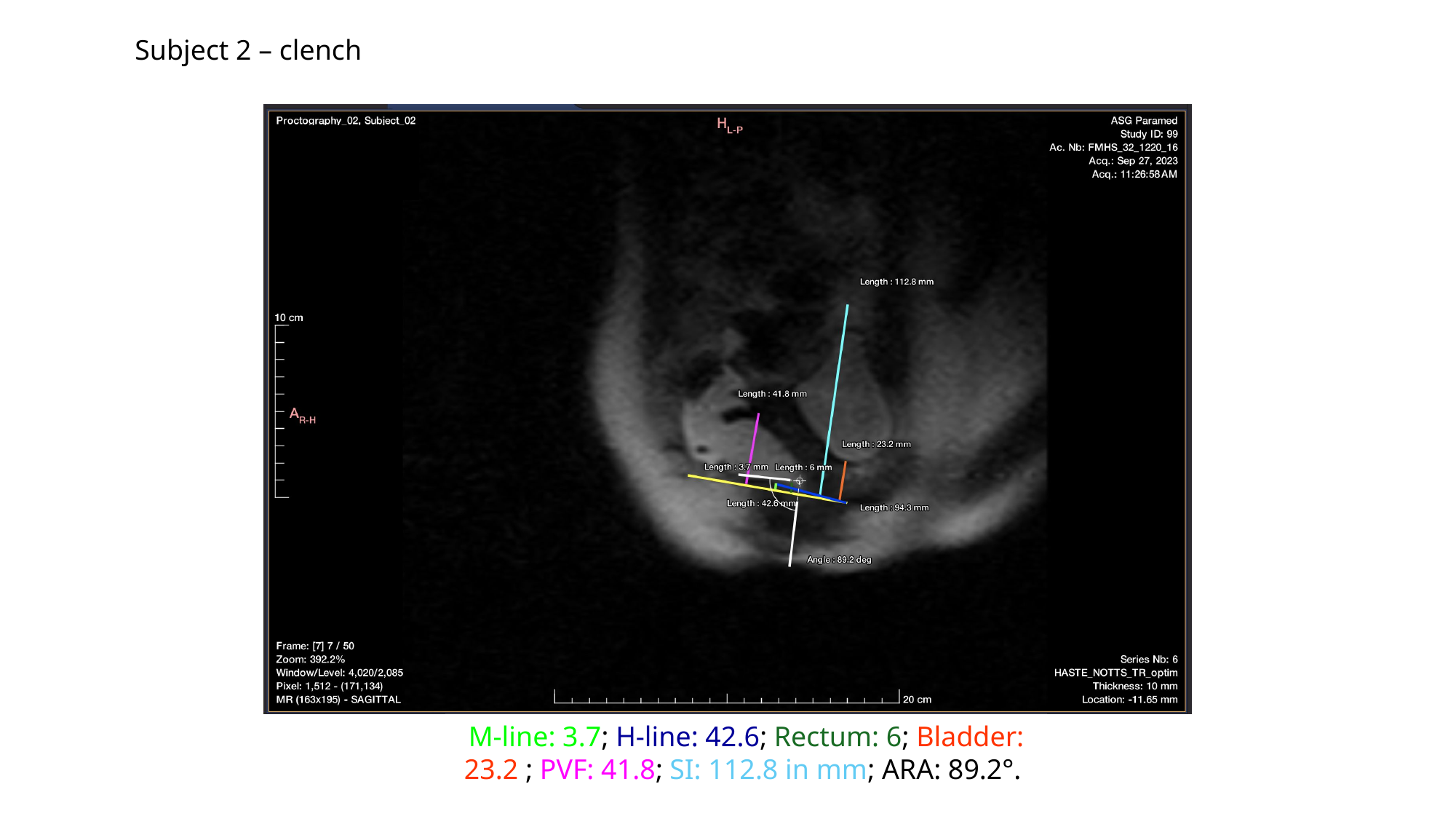

Subject 2 – clench
M-line: 3.7; H-line: 42.6; Rectum: 6; Bladder: 23.2 ; PVF: 41.8; SI: 112.8 in mm; ARA: 89.2°.

## Slide 3
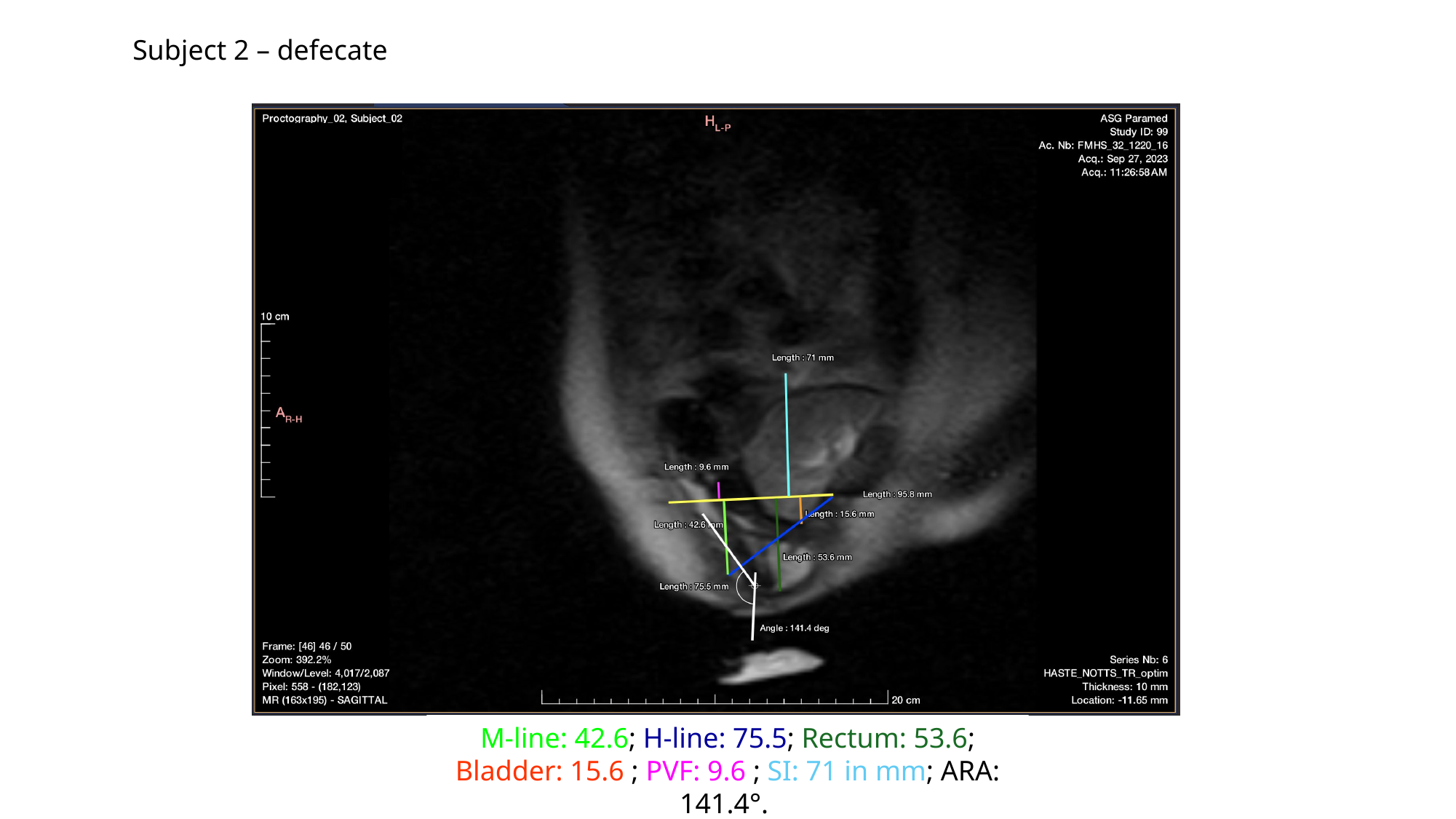

Subject 2 – defecate
M-line: 42.6; H-line: 75.5; Rectum: 53.6; Bladder: 15.6 ; PVF: 9.6 ; SI: 71 in mm; ARA: 141.4°.

## Slide 4
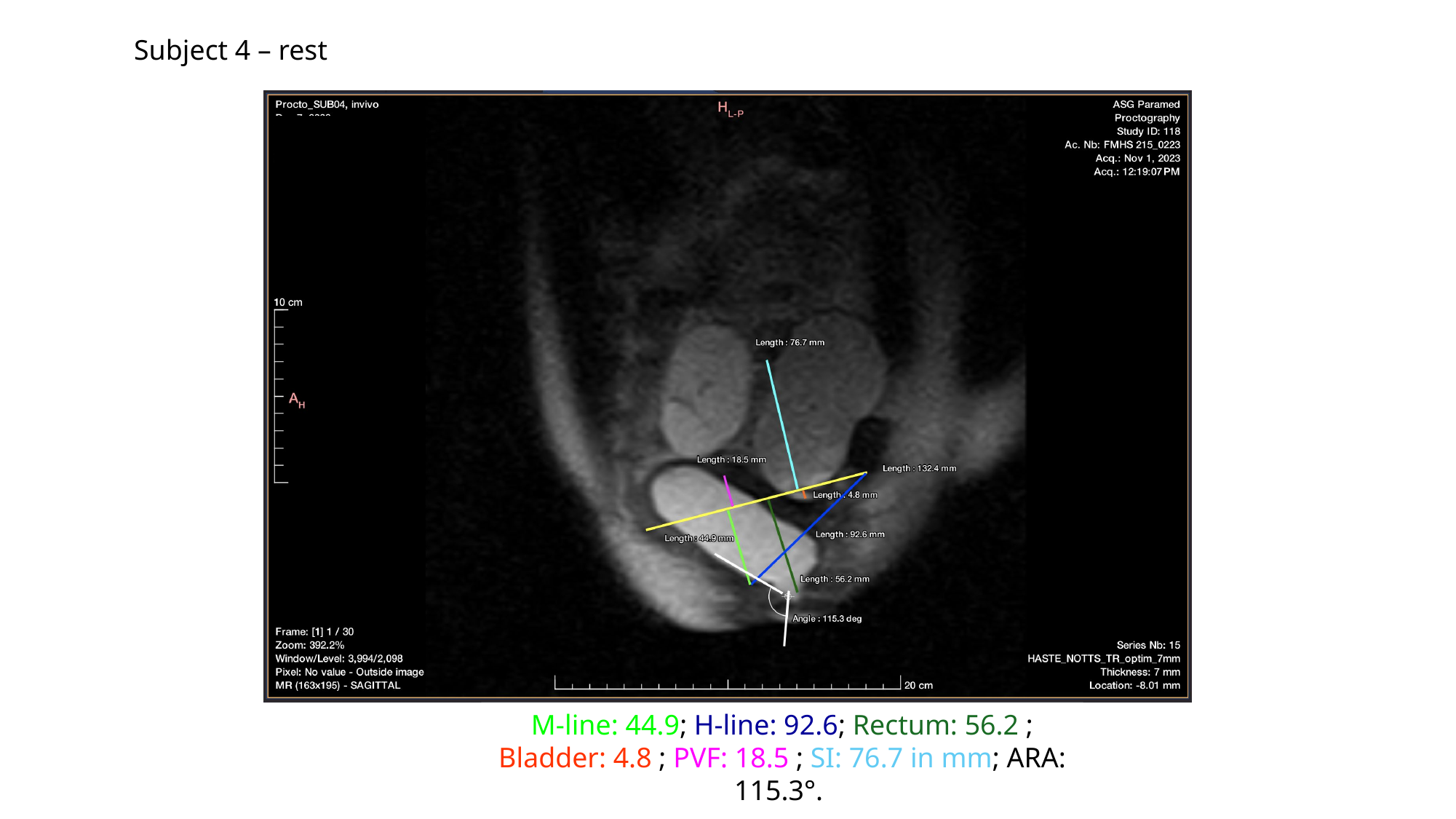

Subject 4 – rest
M-line: 44.9; H-line: 92.6; Rectum: 56.2 ; Bladder: 4.8 ; PVF: 18.5 ; SI: 76.7 in mm; ARA: 115.3°.

## Slide 5
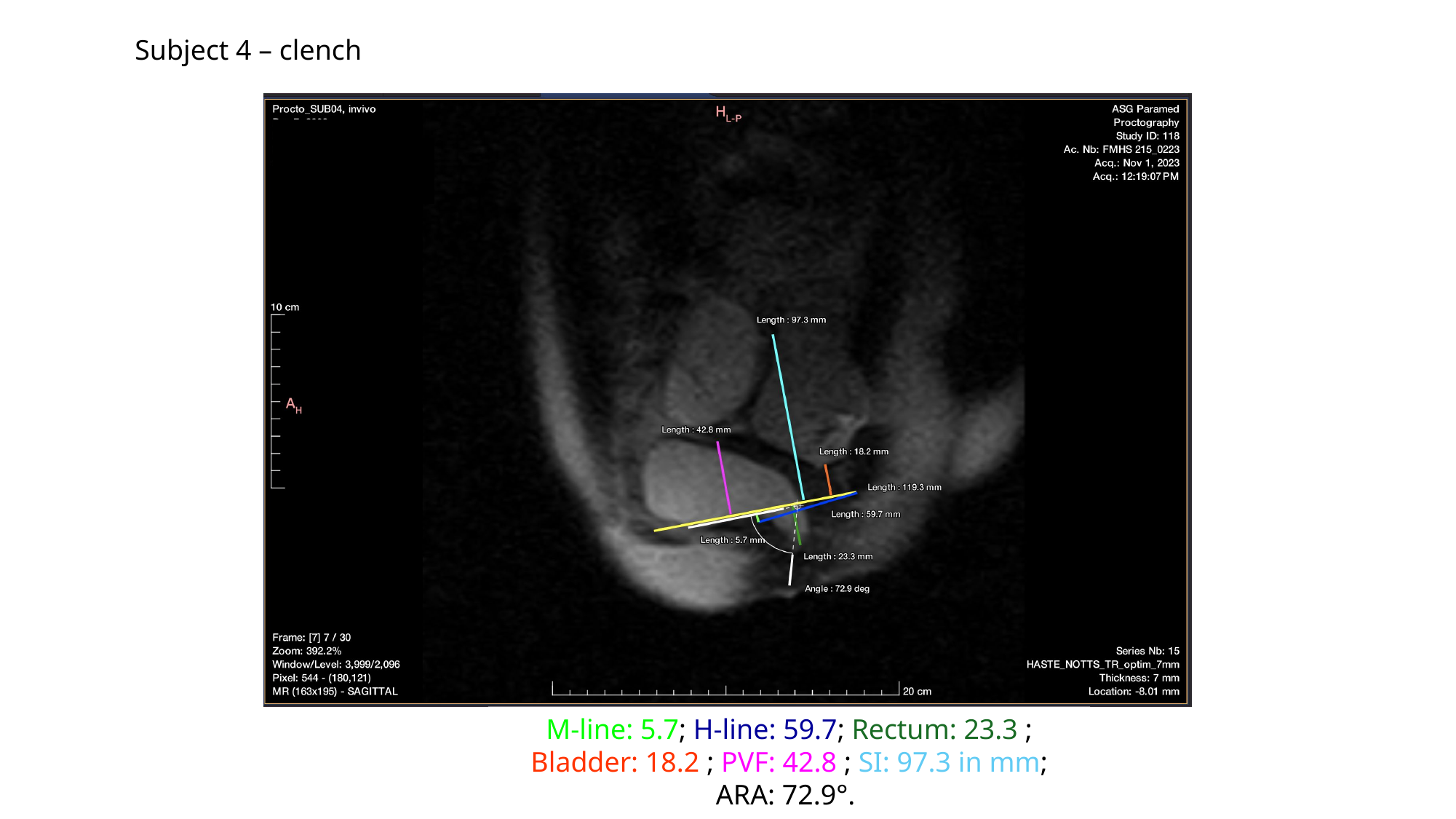

Subject 4 – clench
M-line: 5.7; H-line: 59.7; Rectum: 23.3 ; Bladder: 18.2 ; PVF: 42.8 ; SI: 97.3 in mm; ARA: 72.9°.

## Slide 6
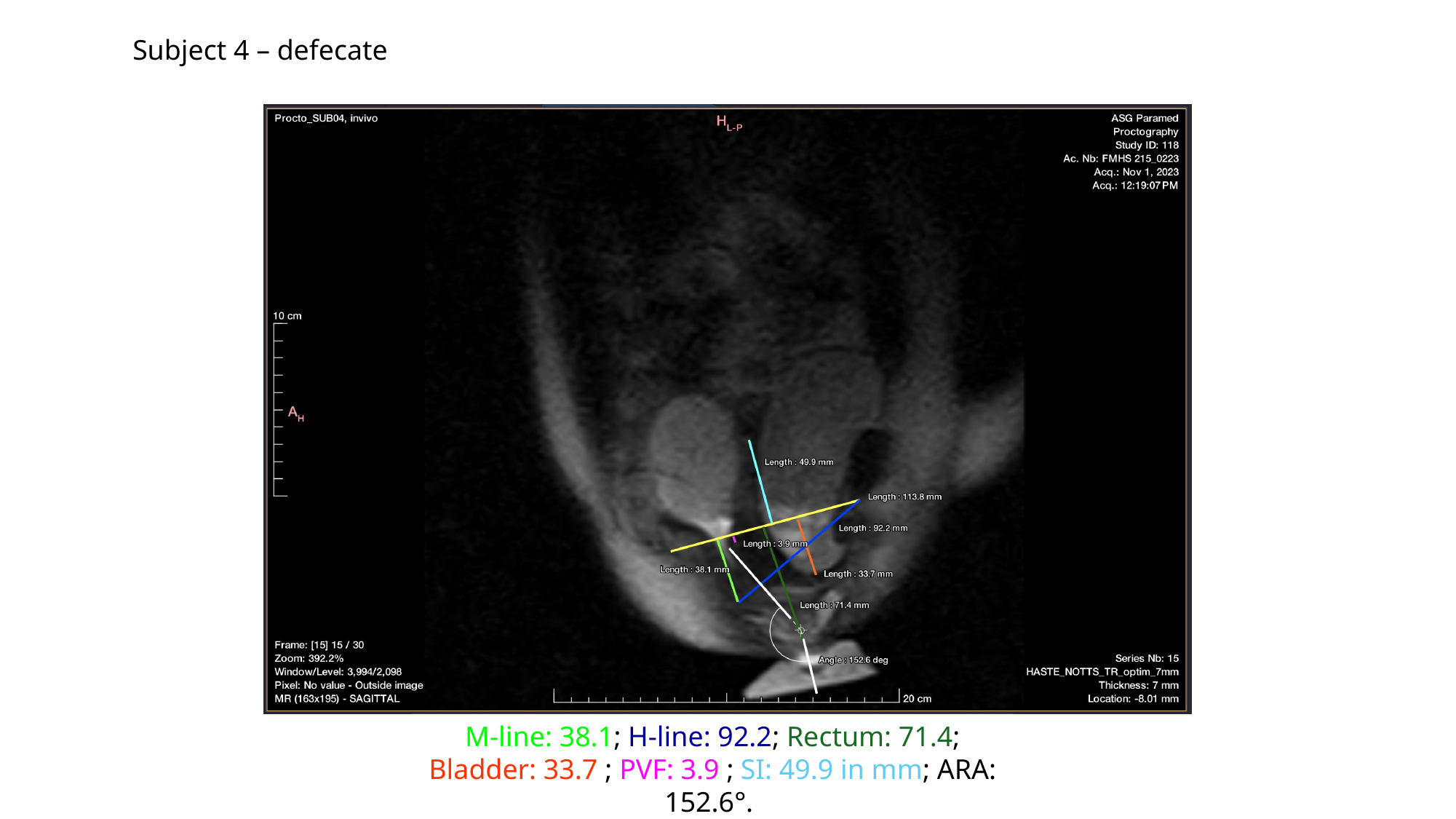

Subject 4 – defecate
M-line: 38.1; H-line: 92.2; Rectum: 71.4; Bladder: 33.7 ; PVF: 3.9 ; SI: 49.9 in mm; ARA: 152.6°.

## Slide 7
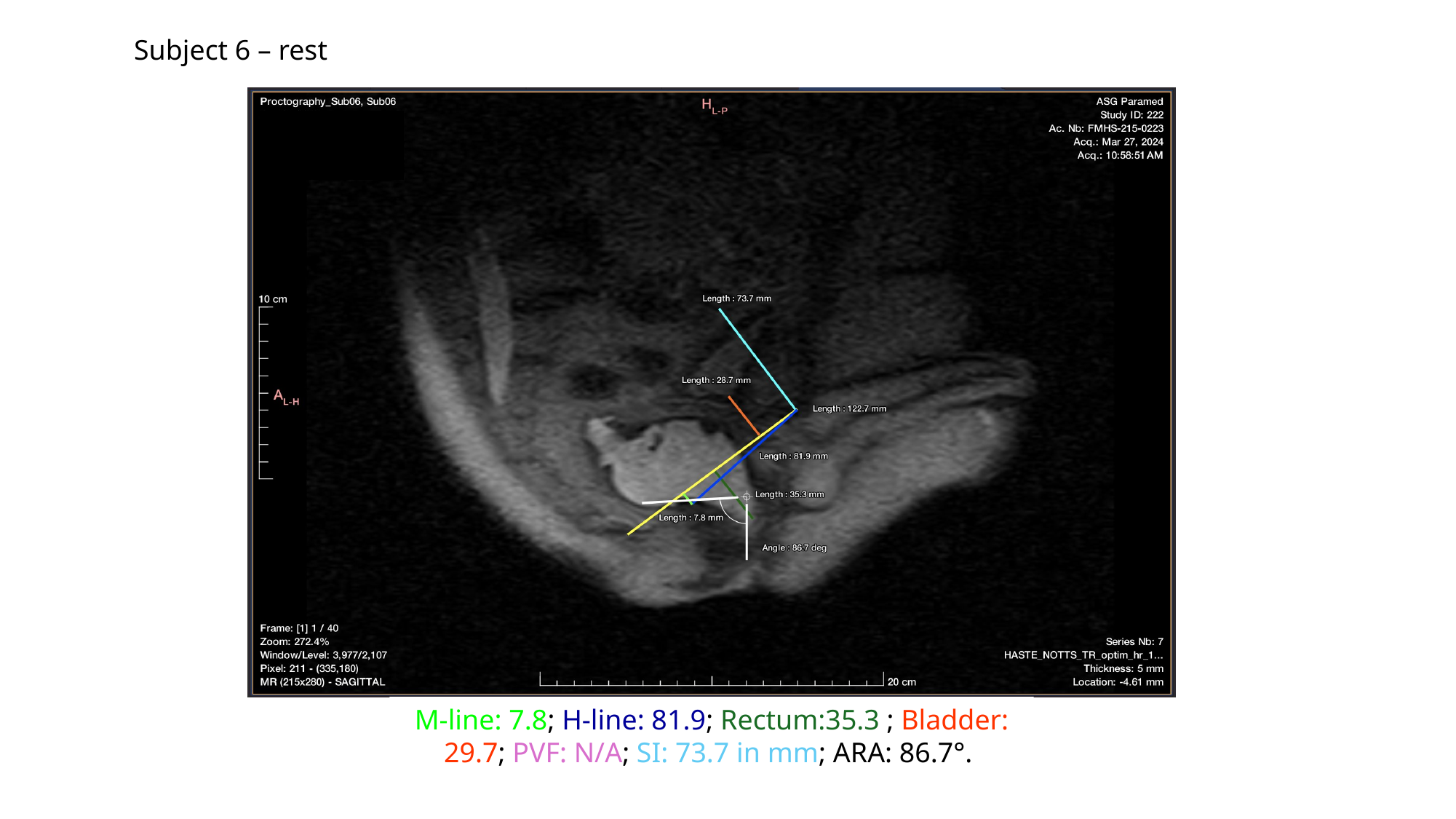

Subject 6 – rest
M-line: 7.8; H-line: 81.9; Rectum:35.3 ; Bladder: 29.7; PVF: N/A; SI: 73.7 in mm; ARA: 86.7°.

## Slide 8
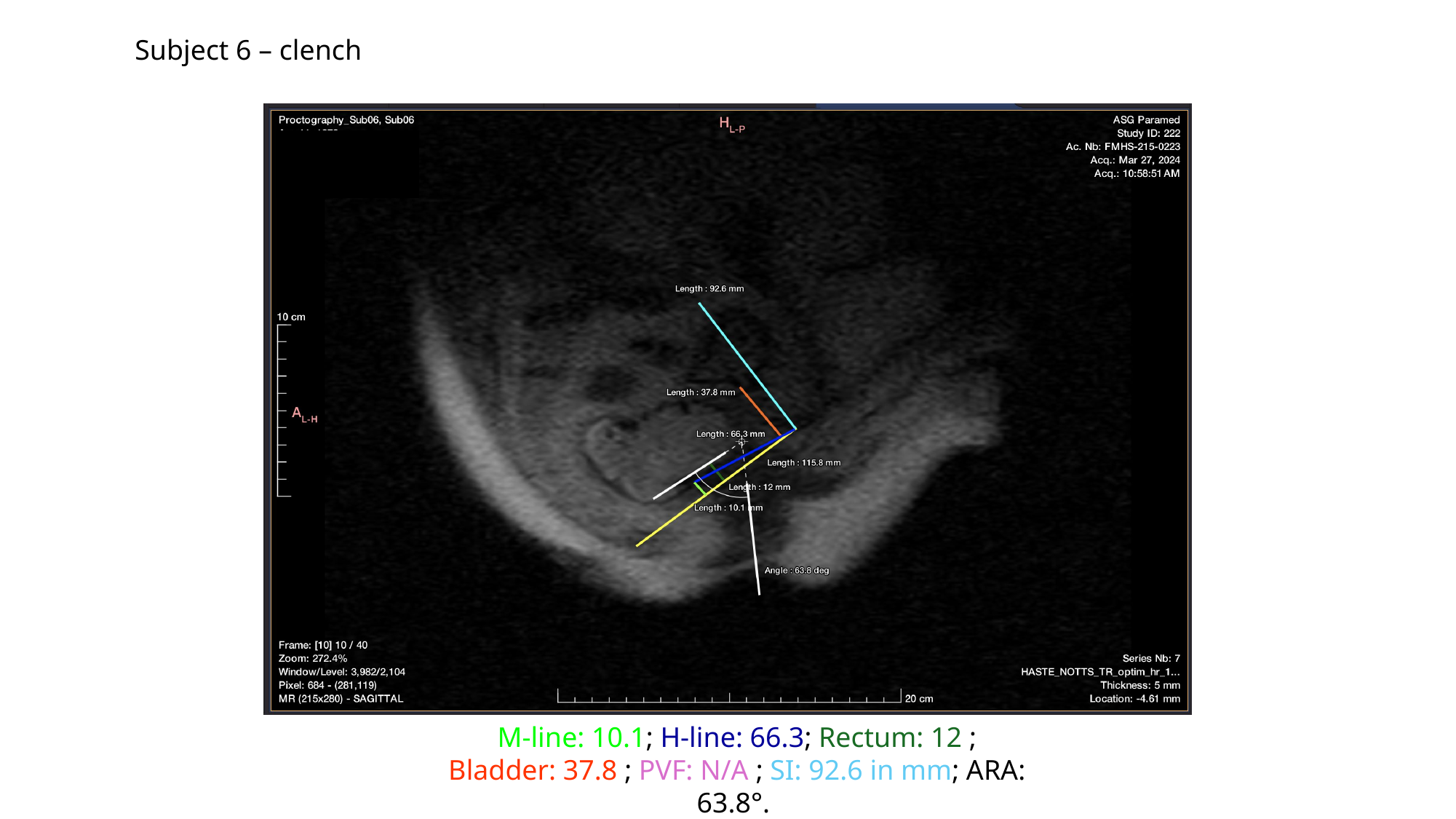

Subject 6 – clench
M-line: 10.1; H-line: 66.3; Rectum: 12 ; Bladder: 37.8 ; PVF: N/A ; SI: 92.6 in mm; ARA: 63.8°.

## Slide 9
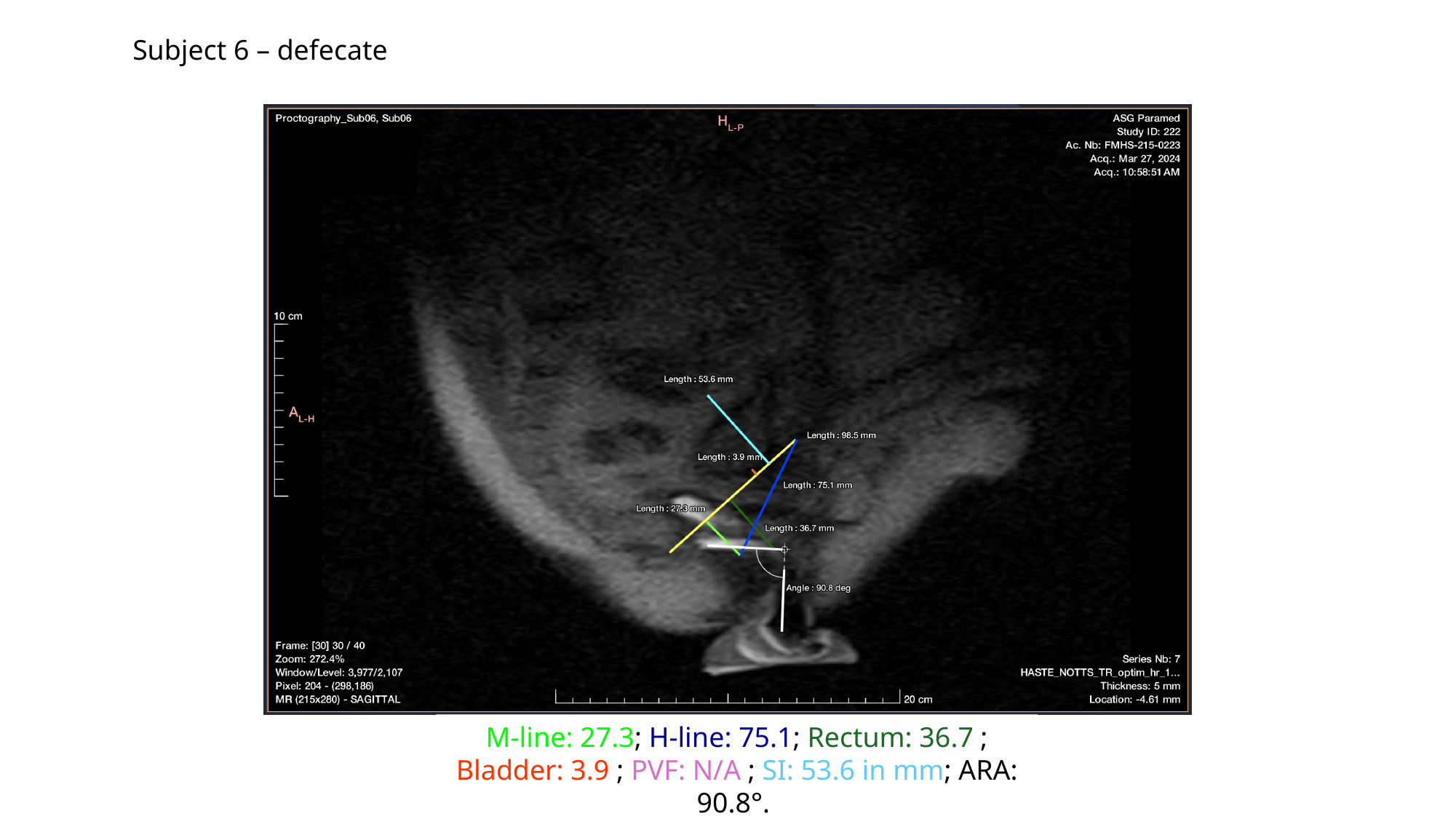

Subject 6 – defecate
M-line: 27.3; H-line: 75.1; Rectum: 36.7 ; Bladder: 3.9 ; PVF: N/A ; SI: 53.6 in mm; ARA: 90.8°.
